# What is hindering Chinese women from participating in combat sports?

**DOI:** 10.3389/fspor.2024.1381895

**Published:** 2024-03-28

**Authors:** Yike Li, Hansen Li, Dongchen Li, Xing Zhang, Zhenhuan Wang, Thomas Green, Guodong Zhang

**Affiliations:** ^1^ Institute of Sport Science, College of Physical Education, Southwest University, Chongqing, China; ^2^ Department of Physical Education and Sport, Faculty of Sport Sciences, University of Granada, Granada, Spain; ^3^ Institute for Health and Sport (iHeS), Victoria University, Melbourne, VIC, Australia; ^4^ Department of Anthropology, Texas A&M University, College Station, TX, United States; ^5^ International College, Krirk University, Bangkok, Thailand

**Keywords:** feminism, women’s combat sports, combat sports, sexual harassment, sports media

## Abstract

With the awakening of female consciousness, women's participation in sports has gradually gained autonomy and agency. However, Chinese women still face numerous restrictions in combat sports, hindering the development of this industry. Based on years of practice and research experience in the field, we summarize some general and specific issues, such as stigmatization and the constraints of traditional Chinese thinking. These issues need attention and consideration in the pursuit of gender equality in sports in the future.

## 1 Introduction

During China's feudal patriarchal era in China, the most common gender division was the principle of “men work outside (home) and women work inside”. This model was achieved through the suppression and denial of women by men (1). During this period, there was a prevailing belief in the superiority of men over women, reducing women to subordinate roles.

After the Opium War (1840–1842) in the Qing dynasty (1616–1912), women began to step out of their homes to participate in social labor, laying the class foundation for the rise of the Chinese women's liberation movement (2). With the emergence of the early women's rights movement in the early days of the People's Republic of China and the ideological liberation brought about by the May Fourth Movement (a significant event in the Chinese New Democratic Revolution), the status of Chinese women was improved. They gained certain rights in areas such as the economy, education, marriage, political participation, and law (3).

Feminism is a political and social movement aimed at advocating for women's equal rights, opportunities, and status. Under the waves of feminist thought and the women's liberation movement, modern women have broken free from past constraints and can, in most cases, freely participate in sports. The international feminist movement, especially after the third wave, has had a significant impact on the inclusion of women's sports events in the Olympic Games (4). Female athletes have started to appear in traditionally male-dominated sports such as judo (5), wrestling (6), and even boxing (7). Since then, female martial artists have gradually entered the public view.

At the end of the 20th century and the beginning of the 21st century, under the influence of Western sports, combat sports such as boxing, Sanda, Taekwondo, kickboxing, and mixed martial arts (MMA) began to rise in China, providing opportunities for female participation (8, 9). However, in the initial stages, female participation was largely limited, and most combat sports were still predominantly male-dominated (10–12). With social progress and the improvement of women's rights, the interest and opportunities for Chinese women to participate in combat sports gradually increased. Some influential female combat athletes, such as Weili Zhang (UFC athlete), began to emerge, inspiring more women to engage in combat sports.

Despite these advancements, most women still face limitations imposed by traditional systems, culture, and other factors. For example, combat sports are often described as “hyper-masculine (13–15)”, leading to stereotypes and biases against women in public perception and media portrayal. This indirectly results in a severe gender imbalance in the participation of combat competitions (16).

Empirically, participation in combat sports enables women to overcome physiological and psychological limitations, enhance self-confidence (17), and face difficulties with greater confidence in other domains. Furthermore, combat sports empower women and grant them autonomy (18), breaking traditional gender roles and demonstrating their ability to achieve remarkable results in the arena. This, in turn, may contribute to changing societal stereotypes and gender perceptions. Given these benefits, we believe that breaking the existing gender barriers and enabling more women to freely participate in combat sports would be of extraordinary significance.

However, it's essential to recognize that China has a unique historical and cultural background, and women may face many unusual challenges when participating in combat sports. Understanding these challenges can provide directions for our efforts toward gender equality in the future. Given the current lack of dedicated articles discussing this topic, we present several perspectives based on conducting combat training and research in China.

## 2 Results and discussion

### 2.1 Influence of traditional Chinese thought

The basic characteristics of traditional Chinese culture include a focus on ethics, advocating moral superiority, emphasizing harmony and unity, pursuing stability, and valuing rationality and humanistic education (19). The Confucian concept of “li” 「礼」 (20) (norms and rituals that individuals should follow in social life) has, to some extent, placed invisible constraints on competition, greatly hindering the development of competitive sports activities (21). For example, during the Western Zhou period (1046-771 BCE), the development of archery rituals excessively adhered to “li”, gradually losing its competitive nature. The Analects of Confucius mentions, “A gentleman has nothing to contend with others; if there is, it must be in archery game. In a game, they bow to each other in courtesy before starting. After the game, they drink together gentlemanly. This is a competition befitting a gentleman” 「君子无所争, 必也射乎!揖让而升, 下而饮。其争也君子」 (22). This emphasizes humility and courtesy, discouraging competition. The Classic of Filial Piety states, “The body, hair, and skin—received from one's parents, one may not injure. This is the beginning of filial piety” 「身体发肤, 受之父母, 不敢毁伤, 孝之始也」 (23). This suggests that everything on the body is a gift from parents and must not be harmed, discouraging risky activities and making people unwilling to engage in dangerous sports (24). The Doctrine of the Mean (a philosophy of moderation) downplays people's competitive awareness. These viewpoints are obviously at odds with the survival principles of competitive sports and have long influenced the Chinese understanding of sports. This is the reason why many Chinese view combat sports as violent and brutal movements activities (25).

Moreover, the traditional Chinese culture characterized by Confucianism has imposed normative requirements on women, reinforcing the patriarchal gender roles of male dominance and female submissiveness. Concepts such as “three obediences (obey father before marriage, husband after marriage, and son after husband's death)” and “four virtues (morality, speech, appearance, and household management)” emphasize male superiority and female inferiority, reinforcing feudal ideas. Confucianism's paternalistic system and ethical views severely restricted the development of women's sports, stipulating that women shall not participate in sports activities, depriving them of the right to sports and stifling their athletic desires. For example, the traditional saying in Chinese martial arts, “Kungfu must be conveyed to male but not to female” 「传男不传女」, fundamentally cut off opportunities for women to participate in martial arts sports (26).

### 2.2 Constraints of societal gender role expectations

Gender in the context of traditional Chinese culture refers to the differentiation of sex, implying a strict boundary between human males and females (27). Social gender, first proposed by feminist Oakley, is a concept relative to biological sex (28), which refers to societal expectations and norms regarding the characteristics, roles, activities, and responsibilities of men and women. Unlike the innate physiological and natural gender, social gender has been shaped by thousands of years of patriarchal society, a result of male-dominated culture (29). Social gender is a mutable, uncertain cultural construct (30), where the thoughts, behaviors, and interactions of men and women are influenced by societal norms, forming an already-established ideology of inequality. This intentional construct confines women, marking their bodies with distinctive symbols, and instructing them to exhibit personality traits deemed appropriate for females. Such constraints prevent them from resisting within the male-dominated environment (31). Consequently, individuals naturally align their cultural behaviors with gender expectations, with men and women respectively playing roles based on societal role expectations. Scholars have found that sports reflect and construct a deeper sense of male superiority and female inferiority than any other social institution (32). The societal construction of women's roles implies that women must participate in specific sports, primarily those emphasizing aesthetic qualities, while most physically confrontational, intense, and high-intensity sports are traditionally considered male domains (33, 34). Women unwittingly internalize these frameworks as psychological biases, leading to compromises and value identification with gender roles. This role positioning, combined with gender differences, influences women's behavioral choices and expressions of interest in sports participation (35). Due to the intense confrontations involved, combat sports have naturally become sports suitable for men. Women participating in sports traditionally deemed suitable for men challenge traditional gender roles and societal expectations (36).

### 2.3 The plague of stigmatization labels

In most cultures, the image of combat clashes with the societal construction of the ideal woman (37), leading to severe stereotypical impressions of female combat sports athletes. For the convenience of training, many female combat athletes keep short hair, becoming one of the most noticeable outward differences from their peers. Additionally, the prolonged training makes them muscular, easily earning them the label of “tomboy” (38, 39). When their achievements surpass those of males, it undoubtedly challenges and undermines the hegemonic status of male sports. Consequently, males often objectify female sports participants in a rejecting manner, then use discriminatory language to cast shadows on women's psychology to solidify their dominance.

Participating in this sport, where direct physical contact is central, female boxers are not only required to enhance physical fitness, skills, and proficiency in using violence but also must display their feminine image outside the boxing ring (40). Otherwise, because of participating in a “masculine” sport (41), they may be labeled as “homosexual” (42), subjected to discrimination and exclusion, and subjected to unwarranted speculation about their sexual orientation. Therefore, to balance the male-dominated world of sports and societal expectations of feminine qualities (43, 44), they adopt apology behavior (45), aligning their appearance and behavior with feminine expectations. For instance, by wearing pink or floral-patterned gloves, competing in skirts, and keeping long hair, they express their feminine characteristics to reduce the risk of being labeled (46).

### 2.4 The worries of “inappropriate contact”

Combat sports involve one-on-one training. In environments typically dominated by males, introducing direct physical contact sports for females may pose challenges (47). To quickly improve their technical skills, athletes need to spar with others having different styles and skill levels. Since female athletes are often scarce in training centers, they may find themselves engaging in sparring sessions with male athletes. Various types of touches are involved in these sparring sessions, some lasting for extended periods. The contact points range from hands, forearms, waist, thighs, to the entire body. Confucian norms such as “men and women should not touch each other” 「男女授受不亲」 (emphasizes that men and women should not be overly close in cohabitation) (48) can lead to widespread gender anxiety, particularly concerning physical contact, when male and female athletes participate in combat sports training. Athletes are concerned about “inappropriate contact”, such as touching someone's genitals, which can hinder some practitioners from focusing on the technical application of combat training (49), thus impeding the effectiveness of their training to some extent.

### 2.5 The excessive “concession” of male sparring partners during training

Combat sports fundamentally rely on live sparring for skill development, where technical proficiency is honed through continuous combat. However, in combat training, male athletes exhibit a tendency to “concession” when facing female athletes (49, 50). Some male combat athletes hold the viewpoint that female teammates lack the endurance to handle training, striking, or grappling at the same intensity as males (51, 52). While male athletes need to display a certain level of self-restraint in most combat training scenarios, such restraint can usually go excessive and become “concession”. On one hand, this excessive “concession” makes it difficult for female athletes to gain rich combat experience, restricting their development. On the other hand, it may make female athletes struggle to find a sense of belonging in the training collective and lead to doubts about their identity as athletes. These negative feelings can further weaken their training motivation and participation. Moreover, if male athletes avoid sparring with females solely based on gender, it may deepen female athletes' awareness of gender discrimination. They might perceive it as an insurmountable barrier, affecting their view of the sport and society, thus hindering female athletes from gaining equal opportunities and treatment.

### 2.6 The burden of losing to male sparring partners

Contrary to the excessive “concession”, another scenario arises in live sparring. When male athletes disregard any gender differences and engage female athletes as ordinary opponents, male athletes consistently gain the upper hand. This sporting experience may potentially reinforce essentialist beliefs about male physical superiority, emphasizing once again the biological differences that may exist between genders (53). Such experiences are likely to dent the confidence of female athletes, leading them to question their own worth. Ultimately, they may become increasingly apprehensive in facing male opponents, resulting in subpar athletic performance and forming a vicious cycle of “fear—poor athletic performance—fear”. This situation is frequently brought up by female athletes in our years of combat training. Once this psychological shadow takes shape, it becomes a bottleneck in training, gradually causing athletes to lose interest in the sport. Such mental health issues for athletes should not be underestimated, as they may impact physical function and athletic performance, and in severe cases, lead athletes to announce retirement (54).

### 2.7 Sexual harassment

The definition of sexual harassment is complex, and commonly understood as gender-themed verbal, non-verbal, or physical behaviors, encompassing any unwanted sexual attention perceived as offensive, intimidating, or humiliating. This form of harassment can be intentional or unintentional, legal or illegal (55). In sports, often considered a male-dominated culture, various forms of discrimination against female athletes are prevalent (56, 57). Research indicates that over 30% of female athletes have experienced sexual harassment in sports (58), with higher proportions in masculinized sports (59). For instance, up to 41% of athletes report experiencing sexual harassment in combat sports scenarios (47). Over the past few decades, numerous news and criminal cases have highlighted the systemic issue of sexual harassment in combat sports (60). Unlike most sports, training with members of the opposite sex is common in combat sports due to the predominance of males. This might lead coaches or fellow athletes to exploit their positions for unnecessary physical contact (such as pinching, hugging, touching) or unwarranted comments on athletes' bodies, attire, and private lives.

Besides training partners, perpetrators of sexual harassment and abuse in sports are often individuals in positions of power (55). A survey once exposed a shocking fact that all interviewees had experienced sexual harassment from male authority figures in the sports industry (61). However, the victims were too afraid to report or fight back due to fear of retaliation or unfair treatment on the training field. The unique ranking system in combat sports, with its strict belt levels (e.g., white belt to black belt), exacerbates the severity of the sexual harassment issue. Athletes show respect and admiration for coaches and higher-ranked teammates. However, this respect can be distorted in some cases, becoming a catalyst for sexual harassment. Many victims have expressed that their respect for coaches or higher-ranked athletes became a factor in their victimization (60).

### 2.8 Inappropriate media reports

Media professionals provide various services to the public (62). However, sports media coverage suffers from severe gender imbalances. Studies show a significant lack of opportunities for reporting on female athletes on platforms like Twitter and similar media (63). In daily reporting, the representation of female athletes in print media is only around 10%, and in broadcast media, it remains below 5% (64). In the United States, despite 40% of sports participation being by women, sports media typically allocates only 5%–8% of coverage to female athletes (65), and similar situations have been reported in other countries as well (66). Furthermore, Gender stereotypes still dominate news content, with sports media tweets more often focusing on the failures of female athletes and belittling their achievements (67). Research revealed that one-third of the tweets implied negative images highlighting the failures of female athletes (68). In such cases, female athletes are often underestimated and underappreciated, with more attention paid to their appearance, clothing, family, and personal relationships than their performance (69).

Media serves the market, providing audiences with what they need. Despite the considerable achievements of female sports, media descriptions of these athletes have long been influenced by objectification and invisibility. Compared to male athletes, female athletes are considered inferior, and media attention to the physical appearance of female athletes far exceeds their focus on their athletic skills and abilities (70, 71). Media sensationalizes female bodies, looks, and related matters to cater to the specific preferences of male audiences seeking “eye-catching stimuli”, attracting public attention. The emphasis on feminine traits such as breasts, buttocks, lips, and nails in sports reporting constructs a “gender hierarchy” in sports (72), marginalizing, trivializing, and devaluing women's athletic achievements. Such an online environment limits gender equality and women's progress. Combat sports provide a typical example. Due to the focus on the male-dominated consumer market, event organizers may host events like “Battle of Beauties”, featuring attractive female athletes, extensively promoting the matches before the event to gain attention. Such practices contradict the original principles of combat sports and place female athletes in a dilemma between pursuing their abilities or appearance.

## 3 Conclusion and future directions

In general, we have presented some perspectives concerning the challenges that Chinese women are facing in combat sports. Here we also make some points for future consideration when trying to cope with these challenges. First, we believe that the Chinese government's policy can play a pivotal role, as they have regulated the sports industries in China for decades. For example, developing some physical education based on combat sports in schools can give female students more chances to understand such sports and foster related interests. Second, we could resort to the “leader effect”. Since some famous athletes such as Weili Zhang have earned great influence in UFC and gradually attracted a number of fans in China, more events in China that they can attend may draw the attention of more women to this sport. Thirdly, we can concentrate on developing some extensions or variants that are relatively easy to play and watch, somewhat similar to flag football (a variant of American football). Such variants can be more friendly for the general female population, thus forming a better foundation for more professional combat sports.

## Data Availability

The original contributions presented in the study are included in the article/Supplementary Material, further inquiries can be directed to the corresponding author.
